# Beyond “Maria”: Charting a Course for Maternal Health Equity

**DOI:** 10.1089/heq.2024.0065

**Published:** 2025-05-16

**Authors:** Wendy Post

**Affiliations:** School of Nursing, George Washington University, Ashburn, Virginia, USA.

**Keywords:** maternal mortality, health inequity, maternal morbidity, preventable deaths, simulation training, maternal morbidity review committees

## Abstract

Maternal mortality and morbidity are enduring public health crises disproportionately affecting Black, Indigenous, Hispanic, and other marginalized populations. This inequity highlights the necessity for a comprehensive, equity-driven framework to address systemic failures within maternal healthcare. Although the Maternal Mortality Review Information Application (MMRIA) provides valuable retrospective insights into maternal deaths, its capabilities must be expanded by integration with real-time interventions. Innovative approaches, including obstetric decompensation scoring tools like the Obstetric Early Warning Score and Maternal Early Warning Score (OEWS) and Maternal Early Warning Trigger systems, are strongly advocated.

These predictive technologies, when integrated into electronic medical records, generate real-time alerts that enable clinicians to proactively mitigate complications before they escalate. Simulation-based training further complements these technologies, immersing healthcare teams in realistic, high-stress scenarios drawn directly from maternal mortality case studies. Such immersive programs effectively address implicit biases, enhance diagnostic accuracy, and foster cultural humility, particularly benefiting marginalized populations.

Additionally, the establishment of Maternal Morbidity Review Committees (MMORCs) is proposed as a critical advancement, enabling multidisciplinary, immediate interventions during acute maternal events. Collectively, these innovations aim to transition maternal health care from a reactive to a proactive model, significantly improving maternal outcomes. Highlighted is the urgency for systemic reforms and data-driven interventions to eliminate inequities, prioritizing prevention, equity, and cultural humility to ensure maternal healthcare is equitable, accesible and inclusive.

## Introduction

Maternal mortality and morbidity persist as critical public health crises, disproportionately impacting Black, Indigenous, Hispanic, and other marginalized populations. The Maternal Mortality Review Information Application (MMRIA, pronounced “Maria”) has emerged as a pivotal retrospective tool for analyzing maternal deaths through in this fight, Maternal Mortality Review Committees (MMRCs).^1^ Each case reviewed through MMRIA serves not only as a dataset but also as an impetus for systemic reform. However, retrospective analysis alone remain insufficient to prevent future maternal deaths; a paradigm shift integrating real-time interventions with retrospective insights is essential.

This article promotes a unified, equity-driven framework leverages innovative technologies to predict, prevent, and respond effectively to maternal health crises. Through the integration of obstetric decompensation scoring tools, immersive simulation-based training, and real-time Maternal Morbidity Review Committees (MMORCs), systemic inequities within maternal healthcare can be effectively dismantled.

## Innovations in Maternal Care: From Retrospective to Real-Time Interventions

### Obstetric Decompensation Scoring Tools

While predictive analytics have transformed critical care settings, widespread adoption within perinatal environments remains limited. Obstetric decompensation scoring tools, including the OEWS and Maternal Early Warning Trigger systems, provide continuous, real-time monitoring of vital signs and laboratory results, detecting subtle yet critical physiological changes indicative of impending complications.^2,3^ Alerts generated by these systems empower clinicians to initiate early interventions, significantly reducing morbidity and improving maternal outcomes.^4,5^ Research consistently confirms the efficacy of these tools in enhancing diagnostic precision and patient safety.^6^

Stanford University’s Obstetric Quality of Recovery-10 (ObsQoR-10), initially designed to evaluate postpartum recovery, provides essential insights into maternal readiness for discharge and ongoing health status, highlighting the value of integrating predictive scoring into routine care.^7^ Similarly, tools validated by the California Maternal Quality Care Collaborative predict severe maternal morbidity by assessing cormorbidities, thus reinforcing the importance of data-driven interventions in maternal care.^5^

### Simulation-Based Training: Preparing for the Unexpected

Technological solutions alone are inadequent; comprehensive training of healthcare teams remains vital. Virtual simulation-based training offers immersive experiences by recreating complex maternal emergencies informed by MMRC data.^8^ Such simulations incorporate scenarios representing the experiences of marginalized populations, emphasizing equity and patient-centered care. Simulation training improves teamwork, diagnostic abilities, and communication during maternal emergencies, including postpartum hemorrhage, hypertensive disorders, and sepsis.^9^ Additionally, these programs effectively address implicit biases, fostering cultural humility and equitable care practices among providers. Hospitals implementing simulation-based education report significant improvements in maternal health outcomes, highlighting the value of realistic, scenario-driven training.^4^

### Real-Time Maternal Morbidity Review Committees

Establishing MMORCs within acute care settings represents a critical evolution in maternal healthcare. In contrast to retrospective analyses, conducted by MMRCs, MMORCs convene multidisciplinary teams- comprising clinicians, social workers, and patient advocates-during active maternal crises, utilizing real-time data to guide immediate interventions.^2^

The proactive approach of MMORCs, combined with scoring tools like OWES, has been associated with substantial reductions in adverse maternal outcomes.^6^

### Implications for Policy and Practice

Equity-driven reforms require hospitals and healthcare systems to integrate predictive scoring tools, immersive simulation training, and MMORCs into standard practice. Policymakers are encouraged to prioritize funding and support these innovations, addressing systemic inequities at their root. Establishing standardized guidelines for these interventions ensures consistent implementation and effectiveness across varied healthcare settings. Additionally, mandated extensions of postpartum Medicaid coverage, improved prenatal care access, and compulsory anti-bias training constitute necessary policy actions to eliminate structural disarities in maternal care.

## Conclusion

 Maternal health crises result not from inevitability but from systemic failures amenable to reform. By employing predictive analytics, comprehensive training, and proactive, real-time interventions, maternal healthcare can be transformed into an equitable and effective system. This vision honors the memory of every life lost and reaffirms a committment to ensuring respectful, preventive, and equitable maternal care for all.

## Abbreviations Used

EMRs: Electronic Medical Records
MMORCs: Maternal Morbidity Review Committees
MMRCs: Maternal Mortality Review Committees
MMRIA: Maternal Mortality Review Information Application
ObsQoR-10: Obstetric Quality of Recovery-10 OEWS: Obstetric Early Warning Score
